# Use of a Deuterated Internal Standard with Pyrolysis-GC/MS Dimeric Marker Analysis to Quantify Tire Tread Particles in the Environment

**DOI:** 10.3390/ijerph9114033

**Published:** 2012-11-08

**Authors:** Kenneth M. Unice, Marisa L. Kreider, Julie M. Panko

**Affiliations:** ChemRisk LLC, Pittsburgh, PA 15222, USA; Email: mkreider@chemrisk.com (M.L.K.); jpanko@chemrisk.com (J.M.P.)

**Keywords:** pyrolysis-GC/MS, internal standard, tire wear, environmental, rubber

## Abstract

Pyrolysis(pyr)-GC/MS analysis of characteristic thermal decomposition fragments has been previously used for qualitative fingerprinting of organic sources in environmental samples. A quantitative pyr-GC/MS method based on characteristic tire polymer pyrolysis products was developed for tread particle quantification in environmental matrices including soil, sediment, and air. The feasibility of quantitative pyr-GC/MS analysis of tread was confirmed in a method evaluation study using artificial soil spiked with known amounts of cryogenically generated tread. Tread concentration determined by blinded analyses was highly correlated (r^2^ ≥ 0.88) with the known tread spike concentration. Two critical refinements to the initial pyrolysis protocol were identified including use of an internal standard and quantification by the dimeric markers vinylcyclohexene and dipentene, which have good specificity for rubber polymer with no other appreciable environmental sources. A novel use of deuterated internal standards of similar polymeric structure was developed to correct the variable analyte recovery caused by sample size, matrix effects, and ion source variability. The resultant quantitative pyr-GC/MS protocol is reliable and transferable between laboratories.

## 1. Introduction

In the last decade, governmental chemical registration regulations and corporate sustainability initiatives have increased the need for reliable analytical methods for assessment of consumer product lifecycles [1,2]. Concurrently, analytical method development efforts have focused on development of improved or new analytical methods for discrete chemicals used to formulate or manufacture consumer products, particularly for substances with low predicted no-effect concentrations (PNECs) [3,4]. However, for products widely dispersed in the environment, improved methods are needed to assess exposure potential.

On-road passenger and truck tires represent a widely dispersed outdoor polymer-based consumer product, with a projected 2015 annual global production demand of 3.3 × 10^9 ^units [5]. Over the service life of a tire, approximately 12% of the mass is released to the environment as tire and road wear particles (TRWP) produced through abrasion of the tires by roadway surfaces [6]. The potential for adverse effects to aquatic species resulting from exposure to TRWP in environmental media has been studied by various investigators [7,8,9,10,11]. With regard to human health, non-exhaust emissions, including tire and brake wear, are recognized as potential contributors to particulate matter (PM) exposure [12,13,14].

TRWP are comprised of elongated particles containing tread particles from tires with mineral incrustations from the road pavement. Our previous research has shown that as a result of the addition of the mineral encrustations, the polymer content of TRWP is about one-half that of the original tread material [15]. The particle size distribution of TRWP dictates that the majority of the mass released onto the roadway surface partitions to the roadside soil and freshwater sediment with a small fraction of TRWP partitioning to the air [6,15]. Given the wide dispersive nature of TRWP, a reliable analytical method is required to accurately quantify the concentration of TRWP in air and sediment attributable to the service life of a tire.

For polymer-based plastic and rubber consumer products widely distributed in matrices including air, soil and sediment, pyrolysis gas chromatography/mass spectrometry (pyr-GC/MS) is an accurate and efficient method capable of providing useful information about the potential for human and ecological exposure during various lifecycle stages. Pyrolysis mass spectrometry methods have been used in industrial laboratories to study polymers for more than 40 years [16]. In pyr-GC/MS, thermal energy is applied to split large molecules into smaller fragments. These characteristic volatile pyrolysis fragments can be used to quantify the concentration of tire tread polymer and total tire tread particulate in environmental samples [17,18]. The thermal decomposition process is accelerated by free radical reactions that occur during bond breaking, and many decades of research have shown that large polymeric molecules fragment in a characteristic manner [19]. In quantitative analysis of polymers, the fragments generated by pyrolysis are readily separated by gas chromatography and identified by a mass selective detector (*i*.*e*., pyr-GC/MS). The fragments of higher abundance are generally monomers, dimers or trimers generated from the decomposition of the polymer backbone.

Pyrolysis based methods are particularly useful for soil and sediment samples because previous research has suggested that butadiene-based polymers including those used in tires may be tightly bound to sediment or difficult to extract by traditional methods due to the reaction with naturally occurring polysulphides in anoxic sediments [17]. To date, applications of pyr-GC to the evaluation of polymers in environmental samples have frequently been qualitative, or when quantitative, have lacked methods, such as the use of standard additions or an internal standard, to account for the effect of matrix composition on the generation rate of pyrolysis products [17,20,21,22]. Pyr-GC analysis of tire polymers in environmental samples has been demonstrated in several studies (Table 1). A qualitative application of pyrolysis GC demonstrating the repeatability and feasibility of pyr-GC for detection of tire rubber in environmental dusts was first published in 1966 [23]. Early research in the 1970s through the early 1990s demonstrated that pyrolytic decomposition and analysis of the tire polymer in particulate or bulk samples could be useful for quantitative determination of tire tread particle (TP) concentration in environmental samples [24,25,26,27,28,29,30]. In the last five years, the feasibility of quantification by dimeric polymer markers has been demonstrated using a mass-selective detector (pyr-GC/MS), however there are questions about how the mineral and organic content of different environmental matrices affect the pyrolysis behavior [31,32,33]. Additionally, non-rubber sources of the monomer compounds in environmental samples are known to positively bias measured concentrations and the use of dimeric markers is preferred [32,34]. Quantification of tire tread in environmental sample in many of the prior applications has been by non-specific flame ionization detectors (FIDs) or has relied on a least one monomeric marker. In addition, external standard calibration, which does not correct for matrix effects, has been the only calibration method used in prior determinations tire wear particulate in environmental samples.

The main objective of this study was to identity or modify an available external standard calibration pyr-GC/MS method for reliable quantification of tire tread in environmental samples. A second objective was to determine the conditions or methodological steps necessary to achieve a tread particulate detection limit less than 50 µg/g dry weight for soil and sediment and less than 0.05% dry weight for air using dimeric pyrolysis markers. Pyrolysis methods are well suited for surveys consisting of many samples because the analysis does not require time consuming sample clean-up or extraction steps, and low part-per-million detection limits are achievable with mass spectroscopy [35]. Obstacles to the formal quantification of polymers in environmental matrices, particularly soil and sediment, have included: (1) the absence of available internal standards, (2) small sample mass for soils or sediments (typically <1 mg), (3) historical use of non-specific detectors, such as flame ionization detection (FID), (4) low specificity of the more abundant monomer fragments, and (5) absence of a defined detection limit. Starting with the initial protocol of Kitamura *et al*. [32] and Harada *et al*. [31], we first evaluated whether an external standard calibration method could be used to reliably quantify tire wear particles in laboratory prepared artificial soil samples, and subsequently developed an internal standard method for tire tread particle quantification by dimeric markers with a formally determined method detection limit. We chose to first evaluate the external standard calibration method based on the use of this method in several studies by several different authors, and the absence of prior examples of an internal standard being used to quantify tire wear particles in environmental samples. 

**Table 1:** Summary of historical applications of pyrolysis-GC analysis to measure the tire rubber content of environmental samples in chronological order.

Study (Year)	Pyrolysis Equipment and Temperature	Analysis	Main Pyrolysis Fingerprint Compounds
Thompson *et al*. (1966) [23]	Platinum-rhodium foil probe pyrolyzer (F & M Scientific Corporation Model 80), 640 °C for 12 s, GC-FID.	Qualitative analysis of roadway dust.	Styrene.
Cardina (1973) [25]	Platinum-rhodium probe pyrolyzer(Hewett-Packard Model 80), 750 to 800 °C for 20 s, GC-FID.	Quantitative analysis of dust fall and tunnel dust accumulation by external calibration in the Akron, OH, USA area.	Dipentene, vinylcyclohexene and styrene.
Cadle and Williams (1980) [24]	Soxhlet extraction with benzene and o-dichlorobenzene. Platinum probe pyrolyzer (Chemical Data Systems Model 190), 850 °C for 10 s, GC-FID.	Quantitative analysis by external calibration of soil samples aged at a roof-top location in the Warren, MI, USA area.	Styrene; isoprene/butadiene; vinylcyclohexene.
Lee and Kim (1989) [27]	Curie-point pyrolyzer (Japan Analytical Industries Model JHP 2), 740 °C for 5 s, GC-FID.	Quantitative analysis by external calibration of airborne PM ≤10 µm collected from street in front of university in Seoul, South Korea.	Styrene; isoprene.
Saito (1989) [28]	Curie-point pyrolyzer (Japan Analytical Industries Model JHP 2), 590 °C for 5 s, GC-FID.	Quantitative analysis by external calibration of roadside dust from Kanagawa, Japan.	Styrene monomer; dipentene.
Kim *et al*. (1990) [26]	Curie-point pyrolyzer (Japan Analytical Industries Model JHP 2), 670 °C for 5 s, GC-FID.	Quantitative analysis by external calibration of total airborne particulate from Hiyoshi, Japan area.	Benzothiazole.
Yamaguchi *et al*. (1995)^ a^ [30]	Micro-furnace pyrolysis (Shimadzu Model Pyr-2A), 600 °C, GC-FID.	Quantitative analysis by external calibration of airborne particulate near highway in Kobe area of Japan.	Styrene; isoprene.
Sakamoto *et al*. (1999)^ a^ [29]	Curie-point pyrolyzer (Japan Analytical Industries Model JHP 22), 670 °C for 5 s, GC-FPD.	Quantitative analysis of total airborne particulate from major local road in Urawa-Tokorosawa area of Japan.	3-Methylthiophene; thiophene; 2-methyhlthiophene ^b^.
Kitamura *et al*. (2007) [32]	Micro-furnace pyrolysis (Frontier Lab AS-1029 Auto-shot sampler), 670 °C, GC-MS.	Quantitative analysis by external calibration of airborne PM ≤10 µm from Shizuoka and Saitama prefecture areas.	Butadiene monomer and dimer; Isoprene monomer and dimer; vinylcyclohexene; isoprene dipentene.
Stein *et al*. (2009) [33]	Thermal desorption at 250 °C, then pyrolysis at 700 to 800 °C, GC-MS.	Quantitative analysis by external calibration and regression model airborne PM_10_ in Weisbaden and Darmstadt areas of Germany.	Dipentene; vinylcyclohexene; phenylcyclohexene ^c^.
Unice *et al*. (2012) [this study]	Curie-point pyrolyzer (Japan Analytical Industries JPS-700 Pyrofoil sampler), 670 °C for 5 s, GC-MS.	Quantitative analysis by internal standard calibration of tread in PM_10_ fraction of air, soil and sediment.	Dipentene; vinylcyclohexene ^d^.

^a^ Abstract, tables and figures presented in English. ^b^ Thiophene derivatives are associated with sulfur cured BR, NR and SBR. The global and temporal stability of this marker has not been verified. ^c^ Phenylcyclohexene is a styrene-butadiene dimeric fragment. ^d^ Butadiene, isoprene and styrene also monitored for qualitative confirmation of polymer presence and relative abundance.

## 2. Experimental Section

### 2.1. Pyrolysis-Gas Chromatography/Mass Spectrometry

Samples were pyrolyzed in a Curie-point ferromagnetic pyrofoil (Table 2). A pyrofoil (JAI Catalog number F670) 9-mm in width was used for analysis of both quartz air filters and solid soil and sediment particulate samples [31,32]. The specimens were added to each pyrofoil boat and the foil was folded using a hand press in preparation for analysis. Peaks were identified based on the known fragmentation, mass spectra and retention time for the target pyrolysis products of natural rubber (NR), styrene-butadiene rubber (SBR) and butadiene rubber (BR). The MS was tuned using perfluorotributylamine (PFTBA) at *m/z* = 69, 212 and 502 prior to each analysis sequence in accordance with the manufacturer’s instructions. Calibration curve reference standards were prepared using isoprene rubber (IR) (IR #2200 JSR Co. Ltd), which is the synthetic form of NR and SBR (SBR #1500 JSR Co. Ltd.). Sample analysis was performed by CERI Laboratory (Tokyo, Japan).

**Table 2:** Summary of pyrolysis-GC/MS conditions.

Stage	Condition	Value
Pyrolysis	Equipment	JPS-700 Pyrofoil sampler (Japan Analytical Industry Co., Ltd.)
		JHS-3 Curie-point pyrolyser (Japan Analytical Industry Co., Ltd.)
	Pyrolysis Temperature	670 °C, 5 s
	Interface Temperature	300 °C
	Sample	20 mg (soil, sediment); 4.5 cm^2^ quartz filter (air)
GC	Equipment	6890 Series (Agilent Techn.)
	Column	J & W DB-5MS [30 m; 0.25 mm I.D.; film 1 μm] (Agilent Techn.)
	Carrier Gas	He
	Injection Temperature	300 °C
	Split Ratio	50:1 (soil, sediment); 10:1 (air filter)
	Oven Temperature	50 °C (hold 5 min); 25 °C/min (heating); 300 °C (hold 10 min)
MS	Equipment	5973 inert (Agilent Techn.)
	Mode	Scan mode
	Mass range	m/z = 35–500

### 2.2. Tread Particle Quantification

Specific marker compounds for tread quantification were selected from among the characteristic fragments generated by the thermal decomposition of the tire tread polymers including SBR, BR and NR. Previous studies have indicated that the most abundant pyrolysis products generated from tread polymers and vulcanization accelerators are styrene (SBR), isoprene (NR), dipentene (NR), butadiene (SBR, BR), vinylcyclohexene (SBR, BR) and benzothiazole (vulcanization accelerator) [19,26,36,37,38]. The monomer markers are generated in high abundance, which increases the likelihood of detection in environmental samples. However, appreciable non-tire sources of the monomers, such as diesel exhaust, which contributes to the styrene marker, can potentially positively bias measured tread concentrations [32,39]. A review of potential pyrolysis markers is provided in Appendix 1. The pyrolysis products isoprene, butadiene, styrene, vinylcyclohexene and the dipentene isomers were selected for analysis based on the abundance of these markers and historical use in quantitative analysis (Table 1). The pyrolysis time and temperature (Table 2) were selected to simultaneously maximize the abundance of these pyrolysis products [27,31,32].

Passenger and tire tread consists of a blend of BR and SBR polymers which both yield butadiene monomer and vinylcyclohexene upon sample pyrolysis. Since the calibration curves were prepared from SBR raw polymer, an adjustment factor was needed to account for the additional contribution of BR to these markers in environmental samples. The market average styrene content for the SBR/BR consumption in tires is 15% [32] reflecting a content of 65% SBR and 35% BR, the styrene content of the 100% SBR 1500 polymer used for the calibration curves was 23.5%. Therefore, an adjustment factor of (1–0.235)/(1–0.15) = 0.90 was used to convert the mass of polymer measured by the vinylcyclohexene marker as SBR 1500 to the mass of SBR + BR polymer in the environmental sample. This adjustment relies on an assumption of an approximately linear increase in the amount of butadiene marker generated with a decreasing percent of SBR in a mixture of SBR/BR, which has been confirmed for SBR contents as low as 10% in mixtures of SBR and BR [40]. To calculate tread concentrations from the measured polymer concentrations, the fraction of SBR in passenger tread, NR in truck tread and SBR + BR + NR in all tread was assumed to be 44, 45, and 50 percent, respectively [41,42,43].

### 2.3. External Standard Calibration Evaluation

To evaluate the ability of pyrolysis-GC/MS to reliably quantify tread in a controlled environmentally representative matrix, artificial soil was spiked with known amounts of synthetically generated tire tread particles. The samples were arbitrarily labeled and the laboratory analyst was not informed of the tread concentration. The methods for the artificial soil preparation are described in detail elsewhere [44]. Briefly, 2-mm sheets of specially prepared passenger tire tread (SBR) and truck tire tread (NR/BR) cured formulations (Bridgestone Corporation, Tokyo, Japan) were cut and cryogenically ground in a Pulva Corporation Model A2 Sizer (Saxonburg, PA, USA). Artificial soil similar to that used in ecotoxicity testing was prepared at Akron Rubber Development Laboratory (Akron, OH, USA) [45,46,47]. The soil recipe consists of quartz (78%), Sphagnum peat moss (2%), and kaolin clay (20%). The soil was spiked with five tread concentration levels of 500, 1,000, 10,000, 25,000 and 50,000 µg/g in the ≤106 µm and 106–212 µm size fractions and aliquots of blank soil were also prepared.

Approximately 20 mg of artificial soil was pyrolyzed using a protocol adapted from the methods presented in Harada *et al*. [31] (Table 2). An external standard calibration was used with standards prepared from dilute solutions of raw IR and SBR in chloroform in amounts between 30 and 180 µg. Uncured rubber was used to facilitate the measurement of µg quantities of the materials. The markers used for quantification in the method evaluation study were styrene (*m/z* = 103, 78), butadiene (*m/z* = 54, 39), and isoprene (*m/z* = 68, 39). Calibration curves were prepared at the beginning of each analysis day in an effort to mitigate changes in the ion source condition. The lower limit of quantification was approximately 550 µg/g or less depending on the type of polymer.

### 2.4. Internal Standard Calibration Method Modification

The protocol used in the external standard method evaluation study was modified to accommodate the use of an internal standard, and to improve the ability to detect the dimeric markers. The purpose of the modification was to decrease the method detection limit, and increase the accuracy and precision of the method by controlling for matrix effects and variable GC/MS efficiency. Calibration curves were prepared by plotting the response ratio as function of the amount ratio. The response ratio is defined as the ratio of the integrated peak area for the molecular marker to that of the internal standard. The amount ratio is defined as the mass of the marker in the sample to that of the internal standard in the sample. The dimeric markers were used for primary quantification and the apparent concentration based on the monomeric markers, styrene, butadiene, and isoprene was also calculated for qualitative comparison to the dimeric markers concentrations.

Deuterated internal standards were obtained from Polymer Standards Service USA Inc. (Amherst, MA, USA) and Polymer Source, Inc. (Dorval, QC, Canada). Each pyrolysis marker was paired with a deuterated polymeric internal standard (Table 3). The mass (µg) of SBR/BR, SBR or NR in the sample was quantified using the calibration curves and the known mass of deuterated internal standard compounds spiked into the sample prior to pyrolysis. The MSD ChemStation software (Agilent Technologies) was used to quantify peak areas of the deuterated internal standards and molecular marker compounds based on the target quantification ions identified in Table 3. All peak integrations were evaluated by an experienced chromatographer, and adjusted as needed for accuracy and consistency. The calibration curves were approximately linear over the calibration range; however, a quadratic calibration model was used for enhanced accuracy over the 400X range expected for the SBR/BR polymer mixture in soil and sediment. Quadratic curve fitting was performed and evaluated against the acceptance criterion of a coefficient of determination ≥0.99.

**Table 3:** Pyr-GC/MS markers and internal standards for soil, sediment and air.

Pyrolysis Marker	Tread Polymer	Pyrolysis marker approximate retention time (RT), target m/z and diagnostic m/z	Internal standard ^a^	Calibration Range for Soil and Sediment	Calibration Range for Air
Dipentene ^d^	NR or IR	RT = 9.7 min	d-PI (1,4-d8) ^a^	IR: 1 to 50 µg	IR: 1 to 50 µg
		m/z = 68, 136	m/z = 76		
Vinyl-cyclohexene ^d^	BR, SBR	RT = 7.7 min	d-PB (1,4-d6) ^b^	SBR: 1 to 400 µg	SBR: 1 to 50 µg
		m/z = 54, 108	m/z = 60		
Isoprene ^e^	NR or IR	RT = 1.8 min	d-PI (1,4-d8) ^a^	IR: 1 to 50 µg	IR: 1 to 12 µg
		m/z = 68, 39	m/z = 76		
Butadiene ^e^	BR, SBR	RT = 1.3 min	d-PB (1,4-d6) ^b^	SBR: 1 to 400 µg	SBR: 1 to 12 µg
		m/z = 54, 39	m/z = 60		
Styrene ^e^	SBR	RT = 8.4 min	d-PS (d8) ^c^	SBR: 1 to 400 µg	SBR: 1 to 50 µg
		m/z = 103, 78	m/z = 111		

^a^ d-PI: Deuterated polyisoprene obtained from Polymer Standards Service USA, Inc. ^b^ d-PB: Deuterated polybutadiene (1,4-d6) obtained from Polymer Source Inc. ^c^ d-PS: Deuterated polystyrene (d8) obtained from Polymer Source Inc. ^d^ Used for quantification of tread concentration. ^e^ Used for qualitative review.

### 2.5. Method Detection Limit

The method detection limit (MDL) was established for each of the tread markers using an U.S. EPA reference method, U.S. EPA 40 CFR Appendix B to Part 136, revision 1.11 [48]. The MDL was defined as the minimum concentration with 99% confidence that the analyte concentration is greater than zero for a reference matrix of clean silica sand based on analysis of seven replicate samples containing SBR and IR polymer. The IR spike concentration was 0.1 µg for evaluation of the isoprene monomer and dimer markers. The SBR spike concentration was 1.0 µg for evaluation of the butadiene monomer, and 0.5 µg for the styrene and butadiene dimer markers. The tread detection limit is dependent on the portion of the air filter or mass of sample pyrolyzed. Polymer detection limits were converted to a tread basis assuming a nominal sample mass of 1 mg for air filters and 20 mg for soil or sediment samples. The target spike recovery and relative standard deviation were 80 to 120% and less than or equal to 20%, respectively.

### 2.6. Quality Assurance

Procedural blanks consisted of analyses of silica sand pretreated with an acid and chloroform wash. Method performance for the internal standard calibration was evaluated by an ongoing midpoint calibration recovery check with an acceptance criteria of less than 20% drift from the theoretical concentration. A calibration verification standard was analyzed before and after each analysis series. The acceptance criterion for the verification standard was a percent drift from the theoretical concentration at the calibration curve midpoint of less than or equal to 20%.

### 2.7. Environmental Sample Analysis

The applicability of the method to environmental samples was evaluated by analysis of a soil sample, air sample, and field duplicate sediment samples. The soil, air and sediment samples were collected from the Kyoto area of Japan (May 25, 2011), the Shiga area of Japan (May 16, 2011) and the Potomac River near the District of Columbia in the United States (April 26, 2011). The soil and sediment samples were collected from surface material by experienced environmental field technicians. The soil sample was collected less than 5 m from a road with a daily traffic load of approximately 20,000 vehicles per day. For the sediment samples, field duplicates were collected to assess the repeatability of the result given the small sample size being analyzed (20 mg) relative to the expected heterogeneity of sediment deposition in river systems.

A PM_10 _air sample was collected over a 24-h period according to a designated United States Environmental Protection Agency standard method (RFPS-1298-126) using the Thermo Fischer Partisol ambient particulate sampler (Model 2000 FRM) with a flow rate of 1 m^3^/h and 47 mm quartz filters. The air sample was collected from the Shiga area of Japan with estimated average daily traffic of 30,000 vehicles per day. The sample was collected less than 5 m from the road near a residential structure and recreational area. Air filters were analyzed without pretreatment except for the normal acclimation for PM_10_ measurement. Soil and sediment samples were oven dried at 105 °C to remove moisture, sieved (<1 mm), and homogenized by mortar and pestle. A soil and sediment sample mass of 20 mg was selected based on a review of the literature which indicated an upper limit for pyrolysis analysis of these matrices of between 5 to 20 mg [21,49]. For the quartz air filters, approximately one-third of the filter was analyzed to allow for archival of the filter for future analysis.

## 3. Results and Discussion

### 3.1. External Standard Method Evaluation

**Figure 1:**
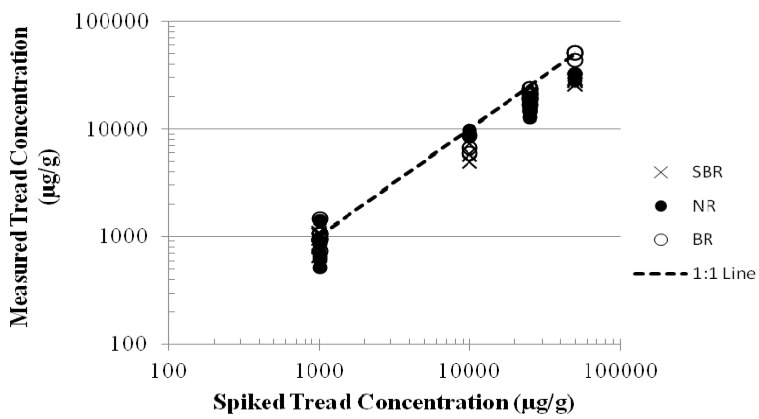
Measured and actual tread polymer concentrations based on pyr-GC/MS analysis of blinded artificial soil samples. Markers are styrene (SBR), butadiene (BR), and isoprene (NR or IR).

Tread particles between a concentration of 1,000 and 50,000 µg/g were detectable by the styrene, butadiene and isoprene markers using the external standard calibration protocol (Figure 1). Actual and measured concentrations were linearly correlated for both size fractions (<106 µm and 106–212 µm) and all three polymer types (r^2^ ≥ 0.88). The average recoveries were 76 ± 16 percent for the styrene marker (n = 21), 71 ± 21 percent for the isoprene marker (n = 21), and 88 ± 18 percent (n = 21) for the butadiene marker. The average relative standard deviation for the monomer markers with triplicate analysis ranged from 9 to 18 percent.

The lower limit of calibration was too high and the impact of changes in the ion source condition was too large for reliable quantification at the 500 µg/g concentration level. It was also observed that when external calibration was used, daily preparation of new calibration curves was required for repeatable results. Quantification by the higher specificity dimer markers (vinylcyclohexene and dipentene) was not possible due to the lower abundance of these marker compounds relative to changes in the ion source condition.

Based on the results of the method evaluation study, pyr-GC/MS was confirmed to be a reliable and appropriate analytical method for tread with regard to specificity and repeatability of the analysis when new calibration curves were prepared each day analyses were performed. However, average recoveries were less than the target range of 80 to 120%, and a need for methodological refinements was identified to achieve a target method detection limit below 500 µg/g. Additionally, modifications were required to allow quantification by the dimeric markers, which have greater specificity for tire rubber polymer than the monomers. These challenges suggested that refinements to the external standard calibration method were necessary before analysis of environmental samples.

During the evaluation of the pyrolysis marker, we also evaluated a less analytically intensive alternate method based on conventional Soxhlet solvent extraction of organic zinc (found as a complex with vulcanization accelerator-related compounds). This evaluation is briefly summarized in Appendix 2. Based on a comparison of the two methods, the quantitative pyr-GC/MS method was determined to be more reliable than the organic zinc method for quantification of TRWP in air, soil and sediment at locations representative of potential human and ecological exposure.

### 3.2. Internal Standard Calibration Method Modification

The failure to reliably quantify the dimeric markers with average recoveries less than 80% in the external calibration evaluation indicated the need for method modifications. Several alternatives to the use of an external standard calibration were considered. For example, Fabbri *et al*. [49] prepared calibration curves with raw polymer in the presence of uncontaminated sediment achieving detection limits based on the benzene and styrene monomers of 0.01 and 1 mg/g for PS and PVC, respectively. However, this method is not suitable for routine use because it does not account for changes in the ion source condition of the mass spectrometer. The use of standard additions was also considered, but was determined to be infeasible for routine analysis given the amount of sample preparation required, limited size of the air filters, and number of analyses required for one sample.

**Figure 2:**
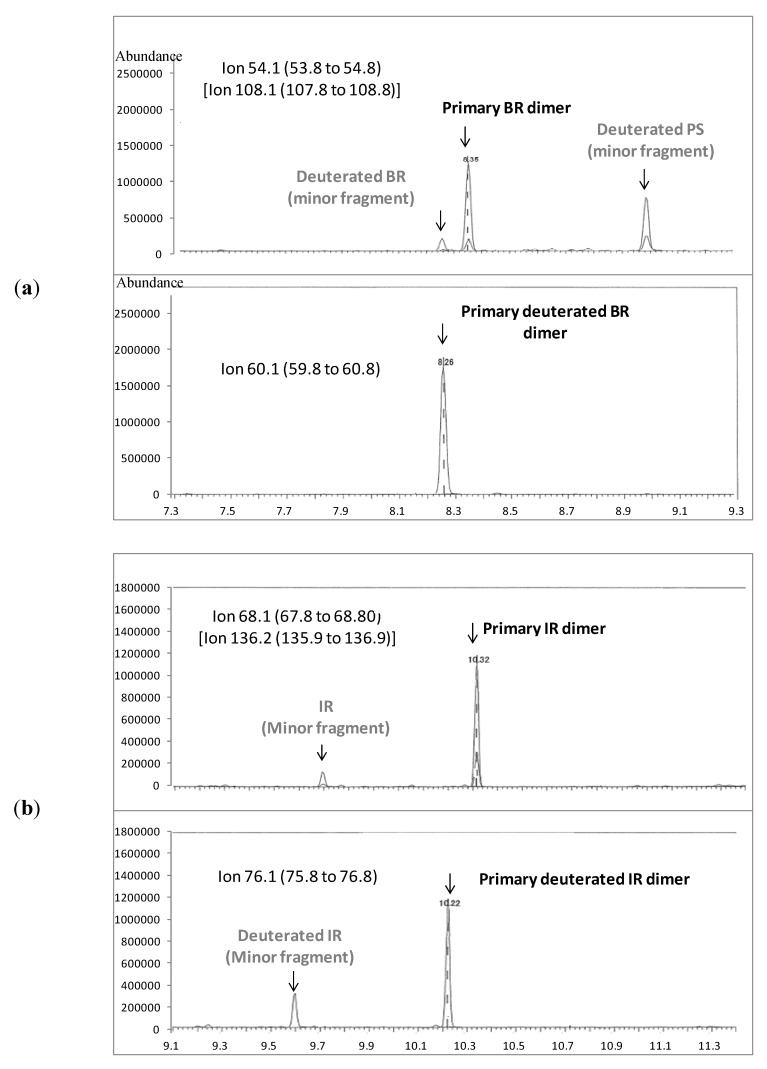
Typical pyrograms generated during calibration curve preparation with identified source. The primary BR and IR dimers are vinylcyclohexene and dipentene, respectively. (**a**) 100 µg SBR with 75 µg deuterated BR; (**b**) 10 µg IR with 10 µg deuterated IR.

After considering other possible approaches, we modified the pyr-GC/MS external calibration method to include the use of an internal standard, which has been frequently applied in conventional GC/MS analysis. Internal standard compounds account for factors affecting fragment generation rates including changes in pyrolysis conditions, sample size, sample aggregation, and sample packing in the pyrofoil [32,50]. The impacts of these factors are corrected by calculating the ratio of instrument response for the target compound to that of specific internal standards spiked into the sample. The use of internal standards is not common in pyr-GC analysis, however some investigators have previously recommended an internal standard calibration procedure to improve the quality of quantitative pyr-GC/MS analysis [50,51,52,53,54].

Our analysis indicated that for the dimeric markers, calibration curves with a coefficient of determination of 0.999 or greater were consistently generated when deuterated (poly)butadiene and deuterated (poly)isoprene were used as the internal standards. Examples of typical internal standard calibration curves for SBR and IR polymer are presented in Appendix 3 and typical pyrograms generated during calibration curve preparation are presented in Figure 2.

### 3.3. Method Detection Limit

**Figure 3:**
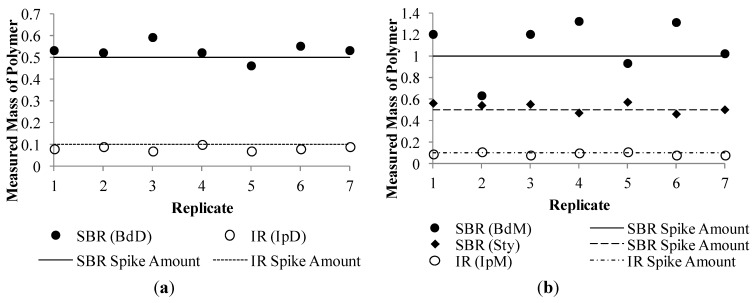
Measured polymer concentration in detection limit study for seven replicates. (**a**) Dimer markers butadiene (BdD) and isoprene (Ipd). (**b**) Monomer markers butadiene (BdM), Isoprene (IpM) and Styrene (Sty).

The method detection limit study was completed following successful preparation of the internal standard calibration curves (Figure 3). The average recovery of measured polymer concentration was 106% and 83% for the butadiene and isoprene dimer markers, respectively, and was 109%, 104% and 93% for the butadiene, styrene and isoprene monomer markers, respectively. The relative standard deviation was 7% and 13% for the butadiene dimer and isoprene dimer markers, respectively, and was 25%, 9% and 15% for the butadiene, styrene and isoprene monomer markers, respectively. The relative standard deviation and average recovery were within the target range with the exception of the result for the butadiene monomer, which exhibited greater variability as compared to the other polymer markers.

For typical conditions, the overall method detection limit for total tread polymer (SBR + BR + NR) is approximately 14 µg/g in sediment or soil particulate and 260 µg/g in the airborne particulate collected on quartz filters (Table 4). These detection limits are sufficiently low to reliably evaluate the fate of TRWP in the environment. Remarkably, with the adoption of an internal standard, the detection limits for the dimeric markers were equivalent or better than the detection limits for the monomeric markers. The dimeric pyrolysis products vinylcyclohexene and dipentene are the preferred chemical markers for tire rubber quantification. Measurment of the styrene, butadiene and isoprene pyrolysis products is useful to confirm the detection of rubber polymer and to qualitatively confirm the magnitude of concentration.

**Table 4:** Method detection limit (MDL) for polymer and tread particulate.

Tire Polymer	Marker	MDL for Polymer (µg)	MDL for Tread in Air (µg/g) ^a,c^	MDL for Tread in Soil and Sediment (µg/g) ^a^
SBR + BR	Butadiene	0.65	650	32
SBR + BR	Vinylcyclohexene	0.10	100	5
SBR	Styrene	0.13	130	6
NR	Isoprene	0.04	40	2
NR	Dipentene	0.03	32	2
SBR + BR + NR ^b^	BdD + IpD	-	260 ^c^	14

^a^ Based on 1 mg of particulate collected on quartz filter and 20 mg of soil/sediment. ^b^ Based on market average SBR/BR and NR use in passenger and truck tread. ^c^ MDL for complete filter analysis. Optionally, 1/3 of the filter may be analyzed and 2/3 of filter is archived for potential future analysis with a resultant detection limit of 780 µg/g for mass of 0.33 mg.

### 3.4. Environmental Sample Analysis

**Figure 4:**
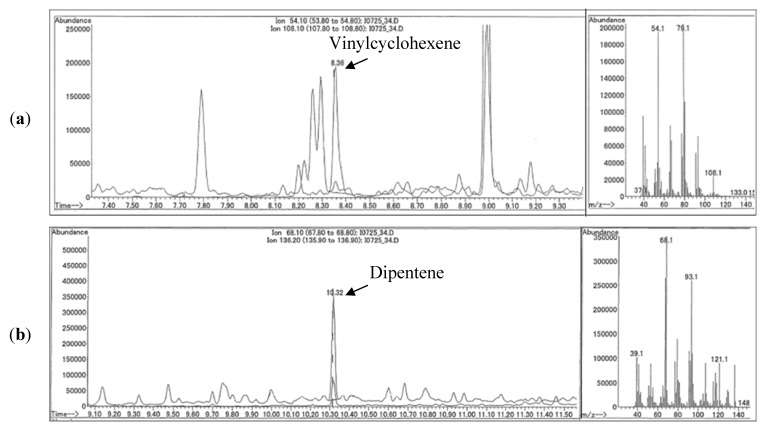
Pyr-GC/MS analysis of a soil sample (longitude, latitude = 34.880222, 135.82389).(**a**) Pyrogram of vinylcyclohexene marker for SBR + BR. (**b**) Pyrogram of dipentene marker for NR.

The application of the method to environmental samples was demonstrated by determination of the tread concentration in one soil and one air sample collected in Japan. For the soil sample, a distinctive peak was detected for both vinylcyclohexene and dipentene (Figure 4). The relative abundance of these markers was confirmed by the detection of styrene, butadiene and isoprene in the sample. The SBR + BR polymer concentration was 850 µg/g dry weight (dw) and NR polymer concentration was 319 µg/g dw. Given a polymer content of tread of approximately 50%, the resultant tread concentration in this sample is 2,300 µg/g dw.

For the air sample, the total PM_10_ concentration was 60 µg/m^3^. Vinylcyclohexene and dipentene were represented by distinct and easily identified peaks (Appendix 4). However, the dipentene concentration was below the method detection limit. The SBR + BR polymer content in PM_10_ was 0.13% and the NR polymer content was <0.0063%. By estimating the NR content as ½ the detection limit and taking the polymer content as 50% of tread, the resultant content of tread particulate in PM_10_ was estimated to be 0.26%. The concentration of tread particulate in air was 0.16 µg/m^3^.

The air sample tread content of 0.26% was consistent with an average tread concentration in PM_10_ of 0.074% to 1.7% in the Shizuoka and Saitma prefectures reported by Kitamura *et al*. [32] for pyrolysis analysis with external dimer calibration with vinylcyclohexene and dipentene. The simultaneous use of both dimer markers for quantification of tire tread particulate in soil samples was not identified in the literature for comparison to our results. Saito *et al*. [28] reported that roadside dust inside and outside of a tunnel near Kanagawa, Japan ranged in concentration from 190 to 2,700 µg/g using an external standard calibration protocol for SBR and NR tread rubber with styrene and dipentene pyrolysis markers, respectively. The concentration of 2,300 µg/g dw observed in our soil sample is between the minimum and maximum concentration reported by Saito *et al*. [28].

The results of the soil and air analysis demonstrate that the dimeric markers for tread polymer can be discriminated and are detectable in environmental samples. There were no appreciable interferences identified for the deuterated internal standards and it can be concluded that matrix effects and changes in ion source condition were taken into account. For both the air and soil sample, the presence of tire polymer in the sample was qualitatively confirmed by the presence and relative abundance of the styrene, butadiene, and isoprene markers in each sample (data not shown).

To evaluate the reliability of the method in sediment given the small sample size (~20 mg) relative to the expected variability of sediment deposition over small (meter) scales, field duplicate grab samples of surface sediments were collected from two different locations of the Potomac river in the Chesapeake Watershed near the District of Columbia, USA (Table 5). At each location, two sequential samples were collected within a 1 m collection area. The relative standard deviation for tread concentration was 15 and 18% for the first and second location, respectively. Analysis of the individual polymer markers revealed greater variability in the isoprene dimer marker as compared to the butadiene dimer marker (Table 5). This result was expected because NR is present in lower amounts closer to the MDL than SBR + BR, and NR is readily degraded under environmental conditions [55].

**Table 5:** Field duplicate analysis of two sediment sample locations (dry weight basis).

Location	SBR + BR Polymer			NR Polymer			Tread		
	First Sample (µg/g)	Second Sample(µg/g)	Relative Standard Dev.%	First Sample (µg/g)	Second Sample(µg/g)	Relative Standard Dev.%	First Sample (µg/g)	Second Sample(µg/g)	Relative Standard Dev.%
Potomac River Location 1 ^a^	266	218	14%	37	29	17%	580	470	15%
Potomac River Location 2 ^b^	27	18	28%	13	9	25%	77	100	18%

^a^ Montgomery County 2 km north west of border of the District of Columbia (longitude, latitude = 39.155100, 77.519091). ^b^ Montgomery County 10 km northeast of Leesburg, Virginia (longitude, latitude = 38.944529, 77.125497).

The field duplicate analysis demonstrates that co-located field samples yield repeatable results. With regard to laboratory duplicate analysis of the same sample, previous pyrolysis analyses of environmental samples in other studies have also revealed acceptable repeatability with relative standard deviations generally 15% or less with sufficient peak area [27,28,49,56]. Despite small sample mass on the order of 0.1 to 10 mg, pyrolysis analyses of air filters and sediments are considered to be reproducible with relative percent differences for laboratory duplicate samples generally about 10% [35]. The relative standard deviation for field duplicate samples was slightly higher in the samples we analyzed, but was still within an acceptable range given the heterogeneity in sub-surface sediment deposition.

### 3.5. Uncertainties in Estimation of Tread Concentration

The use of the pyr-GC/MS protocol described here to quantify tire tread concentration in the environment is not without uncertainty. Use of vinylcyclohexene to quantify the sum of SBR and BR polymer concentration required an assumption about the market share of SBR and BR in tread. However, the validity of this assumption was confirmed by an informal survey of tire manufacturing companies and uncertainty in this value is not expected to appreciably affect the quantification of tread. In addition, minor sources of vinylcyclohexene or dipentene attributable to non-tire polymers may be present in environmental samples resulting in a positive bias in the derived environmental tire tread concentration. However, appreciable alternative sources of these dimers in sample pyrolysis have not been identified (Appendix 1). Finally, the addition of tire tread additives such as anti-oxidants and accelerators varies by manufacture; however, variability in these additives is not expected to affect the pyrolysis behavior of the polymer chains [18].

## 4. Conclusions

A pyr-GC/MS external standard calibration method for rubber polymer was successfully modified to include the use of a deuterated internal standard for measurement of the concentration of tire tread particulate in air filters, sediment and soil. The tread detection limits for the modified method using dimeric markers with specificity for the rubber polymers SBR and BR were approximately 14 and 260 µg/g dry weight for sediment and air particulate, respectively. We concluded that an external standard calibration protocol could not be used to reliably quantify polymers in environmental samples because of the matrix effects on the pyrolysis products and difficulty in reliably quantifying the dimeric marker, which are often lower in abundance than the monomeric markers. The pyr-GC/MS method described here is expected to be useful for assessment of the distribution of TRWP in the environment and subsequent assessment of ecological and human health risk, however, further application of the method to environmental samples is necessary to fully evaluate the method.

## Appendix 1. Review of Pyrolysis Markers for Tread Particulate

Sources of the styrene pyrolysis product include humic substances, vegetables and fruit tannins, degraded lignins from wood and paper, plastic wastes and diesel exhaust [17,39,56,57]. The monomer 1,3-butadiene is a component of vehicle exhaust formed from combustion products of olefins, alkanes and aromatic compounds [58]. In pyrolysis of environmental samples, five carbon fragments generated from natural organic matter are likely to interfere with isoprene [32].

The dipentene and vinylcyclohexene fragments are specific to a polymeric source in part due to the abundance these compounds generated in rubber pyrolysis as compared to other potential polymeric sources [32,59]. The primary analytical challenges with dimeric markers are the potentially lower abundance of these markers as compared to the monomeric markers and identification of a suitable internal standard. Pyrolysis of polyisoprene generates three major dimer species, however at temperatures typical of environmental sample pyrolysis less than 800 K [19], dipentene is the dominant dimer species [36]. Dipentene consists of a mixture of stereoisomers. The right-handed isomer, (+)-limonene, is found in citrus fruits and used in cleaning products and cosmetics. In pyrolysis analysis of rubber traces generated in tire skidding, the dipentene (mixture of isomers) pyrolysis fragment and (+)-limonene eluted at different retention times [60] and limonene is readily biodegradable [61], thus reducing the likelihood of interference from industrial or natural sources of (+)-limonene.

With respect to vinylcyclohexene used to quantify BR and SBR, the primary source in environmental samples is SBR from tire wear [25]. Vinylcyclohexene was not identified as one of the 36 most abundant fragment compounds detected in the humic acid or humin of an Italian agricultural soil [56]. Vinylcyclohexene is also a pyrolysis product of is also a pyrolysis product of acrylonitrile-butadiene-styrene copolymer (ABS) [49], however, wide dispersive release of this polymer is expected to be negligible based on its use in thermoplastic products such as appliances, pipes and shower heads [62]. Data presented by Choi [37] suggests that the generation rate of vinylcyclohexene may increase with the content of silane coupling agent in silica tires such that the concentration of tread measured by this marker compound may be biased slightly higher than the true environmental concentration. In contrast, Groten *et al*. [50] identified pyr-GC as an appropriate method for quantitative analysis of carbon-black containing polymers and Matheson *et al*. [63] showed that the presence of carbon black does not affect the generation of SBR or NR pyrolysis products.

Benzothiazole-related compounds were also considered as potential marker compounds. Benzothiazole pyrolysis products are associated with cured tread rubber through the use of benzothiazole-derived compounds as vulcanization accelerators. However, benzothiazoles are also widely used in industrial processes, and unknown household consumer products were recently identified as an important source of benzothiazole release independent of releases to surface water from rubber tires [64]. Benzothiazole-derivative compounds are also used in antifreeze for automobile radiators, corrosion inhibitors in paper production, intermediates in die production, and fungicides in leather and wood preservation [64,65,66,67]. Furthermore, benzothiazole compounds leach from rubber exposed to the environment over time [66], decreasing the reliability of this compound class as a marker. The low abundance and potential variability of benzothiazole eliminated this marker from consideration in the analysis. Therefore, isoprene, butadiene, styrene, vinylcyclohexene and the dipentene isomers were selected for analysis.

## Appendix 2. Method Evaluation of Organic Zinc Marker

During the method evaluation study, a published organic zinc method was evaluated in parallel with the pyr-GC/MS method [68]. This method was evaluated because it is representative of more simplistic and conventional laboratory analysis techniques including solvent extraction and total metal analysis. The organic zinc method is based on a conventional dichloromethane Soxhlet solvent extraction of organic zinc (found as a complex with vulcanization accelerator-related compounds) followed by quantification of total extracted zinc. Organic zinc was proposed more than 10 years ago as a tread marker based on the occurrence zinc-vulcanization accelerator complexes extractable in dichloromethane, such as zinc-mercaptobenzothiazole. Since development, the method has been used in a few applications [68,69].

Artificial soil samples were prepared by the method described in Section 2.1 and the methods for the organic zinc analysis are described in more detail elsewhere [44]. Briefly, for the analysis, 1 g of artificial soil and 0.1 g of synthetic tread particulate was extracted with 80 to 100 mL of dichloromethane (DCM) in a Sohxlet extractor (ACE Glass Inc. Part # 6716, 125 mL bottom flask) with extraction times varying from 4 to 48 h. The extraction solvent was evaporated and final extract dissolved in nitric acid for analysis of the DCM extracted zinc as inorganic zinc by high resolution ICP/MS. The lower limit of quantification was 0.3 µg organic zinc/gram sample and all analyses were performed at MPI Research (State College, PA, USA).

The concentration of organic zinc was correlated with SBR content (r^2^ = 0.71) and NR content (r^2^ = 0.77) when tread concentrations of 500, 1,000, 10,000, 25,000 and 50,000 µg/g were analyzed. Each concentration level was characterized by repeated analysis (n = 3 to 12) but the relative standard deviation was highly variable ranging from 4% to 54%. The recovery of organic zinc from SBR particulate in soil was very low (<15%). ZnO is not soluble in DCM, however, when it was spiked into the DCM solution to evaluate potential positive bias from inorganic sources, a fine colloidal ZnO suspension was observed. The suspension could not be separated from the solvent by centrifugation or filtration and it was not possible to reliably quantify potential bias from inorganic zinc using a ZnO spike addition. Further study of the organic zinc marker was not conducted based on the lack of a feasible internal standard for this method to account for the very low recovery of organic zinc from SBR in soil and because a reliable method to exclude colloidal ZnO from the DCM extraction could not be identified.

## Appendix 3. Representative Calibration Curves for Internal Standard Method

Typical calibration curves for dimeric markers with deuterated internal standard. (**a**) 1–50 µg SBR with 7.6 µg deuterated BR and 1–12 µg IR with 10 µg deuterated IR for air. (**b**) 1–400 µg SBR with 76 µg deuterated BR and 1–50 µg IR with 10 µg deuterated IR for soil or sediment.

## Appendix 4. Pyrograms for Air Analysis

Pyr-GC/MS analysis of an air sample (longitude, latitude = 35.2826111, 136.250528). (**a**) Pyrogram of vinylcyclohexene marker for SBR + BR. (**b**) Pyrogram of dipentene marker for NR.
